# ARTEMISIA: a mechanistic study of a novel Janus kinase 1 inhibitor to advance molecular understanding and precision medicine in asthma

**DOI:** 10.1186/s12931-025-03309-3

**Published:** 2025-07-02

**Authors:** Davinder Paul Singh Dosanjh, Erika Darrah, Tina Jellesmark Jensen, Zala Jevnikar, Alese Halvorson, Mathias Cardner, Rod Hughes, Sarah S. Grant, Adam Platt, Chris E. Brightling

**Affiliations:** 1 Clinical Development, Respiratory and Immunology, BioPharmaceuticals R&D, AstraZeneca, Cambridge, UK; 2 Birmingham Acute Care Research, Institute of Inflammation and Ageing, University of Birmingham, Birmingham, UK; 3 Translational Science and Experimental Medicine, Research and Early Development, Respiratory and Immunology, BioPharmaceuticals R&D, AstraZeneca, Gaithersburg USA; 4 Clinical Development, Respiratory and Immunology, BioPharmaceuticals R&D, AstraZeneca, Gothenburg, Sweden; 5 Translational Science and Experimental Medicine, Research and Early Development, Respiratory and Immunology, BioPharmaceuticals R&D, AstraZeneca, Gothenburg, Sweden; 6 Biometrics and Statistical Innovation, Respiratory and Immunology, BioPharmaceuticals R&D, AstraZeneca, Gothenburg, Sweden; 7 Clinical Development, Respiratory and Immunology, BioPharmaceuticals R&D, AstraZeneca, Boston, USA; 8 Translational Science and Experimental Medicine, Research and Early Development, Respiratory and Immunology, BioPharmaceuticals R&D, AstraZeneca, Cambridge, UK; 9 Institute for Lung Health, Leicester NIHR BRC, University of Leicester, Leicester, UK

**Keywords:** Asthma, Janus kinase 1, Gene signatures, TH17, Interferon-gamma, Interleukin-6, Signal transducer and activator of transcription (STAT), T2, Inflammation, Bronchoscopy

## Abstract

**Background:**

Patients with uncontrolled asthma despite the use of inhaled corticosteroids (ICS), may have a variety of biological pathways driving their airway inflammation. Londamocitinib (AZD4604), a selective, inhaled, Janus kinase 1 inhibitor, has been designed to target a broad inflammatory cytokine profile including those classically unresponsive to ICS. The ARTEMISIA mechanistic study aims to provide a clear understanding of the pathways impacted by londamocitinib in the lung, determine how this impact is reflected in the nose and periphery, and identify candidate biomarkers of londamocitinib-treatment response in asthma. This article reports the design and objectives of the ARTEMISIA study.

**Methods:**

ARTEMISIA is a placebo-controlled, double-blind study of adults with moderate-to-severe asthma aiming to assess the effects of inhaled londamocitinib on Type 2 (T2) and non-T2 driven inflammatory pathways. Extensive parallel bio-sampling of the lung target tissue, nasal mucosa, blood and urine will be performed prior to the first dose and after 4-weeks of treatment with either londamocitinib or placebo. The main objectives of the study are to evaluate the effect of londamocitinib on gene expression in endobronchial brushings and signal transducer and activator of transcription (STAT) phosphorylation in endobronchial biopsies. Key exploratory objectives include investigating the correlation between inflammatory phenotype-specific bronchial epithelial gene signatures and other biomarkers in the lung and peripheral samples; as well as analysis of transcriptomic, proteomic, and metabolomic biomarkers in the nose, blood, and urine.

**Discussion:**

ARTEMISIA commenced recruitment in 2024 and is poised to deliver a deep understanding of the mechanism of action of londamocitinib and its potential to impact on a population of asthmatics with high unmet need. The multiomic analysis of paired central and peripheral samples may reveal novel insights into the connection and translation between these compartments, deepen understanding of airways disease, and identify novel candidate biomarkers for asthma and JAK activity. In addition to sampling the airway directly, with parallel nasal and peripheral bio-sampling mirrored by the Phase 2a AJAX study (NCT06020014), the ARTEMISIA study may provide a unique link between bronchial assessed mechanisms of action and clinical outcomes.

**Trial registration:**

NCT06435273 (ClinicalTrials.gov). Registered 24th May 2024.

## Background

Asthma is a chronic respiratory disease typified by variable symptoms and airflow limitation related to complex underlying airways inflammation [1]. Whilst for several decades the mainstay of treatment for asthma has been inhaled corticosteroids (ICS) with or without long-acting beta agonists (LABA), the emergence of targeted biologic therapies has driven a new wave of asthma phenotyping and a deeper understanding of its pathobiology. Most current biologic approaches are directed to T2-high cytokine (e.g. Interleukins [IL] 4, 5, and 13) and epithelial alarmin (e.g. thymic stromal lymphopoietin [TSLP], IL-33) based mechanisms which overlap significantly with those pathways that are corticosteroid sensitive [2]. In fact, many clinical trials of these agents have been specifically directed to those patients whose only option has been systemic corticosteroids [3]. However, it is becoming increasingly apparent that many individuals with asthma have at least a component of co-existing non-T2, corticosteroid insensitive airways inflammation (e.g. IL-6, IL-17 and neutrophilic) that continues to drive poor asthma control even after all currently available approaches have been exhausted [4]. There is an urgent need to develop new therapies that target inflammation beyond the classic, predominantly T2 phenotype [1].

**Fig. 1:**
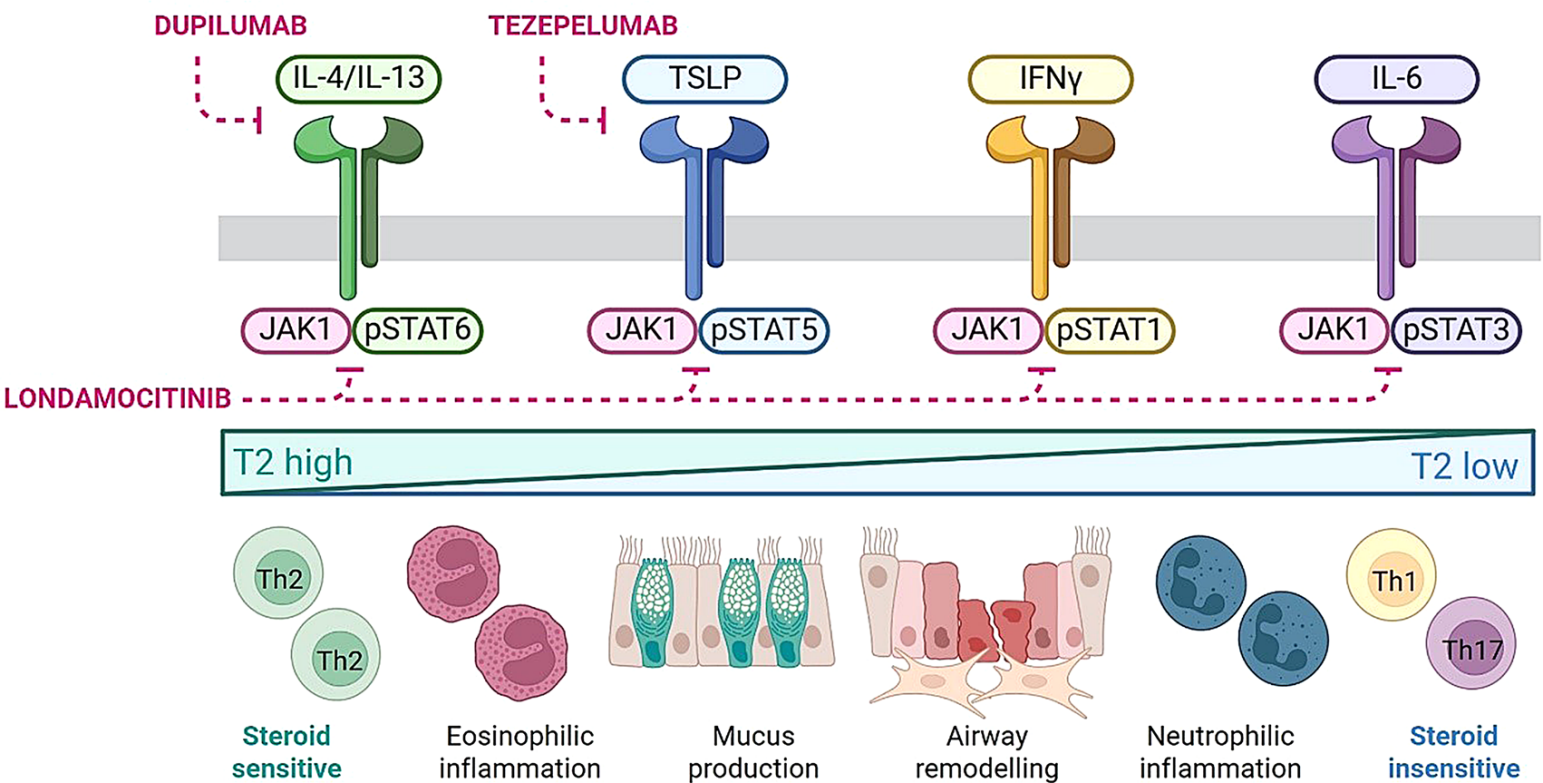
Proposed mechanism of action of londamocitinib. Janus kinase 1 inhibition reduces STAT1/3/5/6 phosphorylation, suppressing inflammatory pathways relevant in asthma, with broader effects than single cytokine biologics. Londamocitinib, a selective JAK1 inhibitor, therefore, has the potential to benefit patients with asthma, including those with steroid-insensitive inflammation, by targeting T2 and non-T2 airway pathology. IFN: Interferon; IL: Interleukin; JAK1: Janus kinase 1; pSTAT: phosphorylated signal transducer and activator of transcription; T2: Type 2; Th: T-helper; TSLP: thymic stromal lymphopoietin. Created with BioRender.com

Londamocitinib (AZD4604) is a highly selective Janus kinase 1 (JAK1) inhibitor that is being developed as a novel inhaled therapy in patients with uncontrolled asthma despite ICS use [5–7]. It exhibits broad inhibition of signalling induced by JAK1-dependent cytokines associated with the entire spectrum of inflammatory phenotypes observed in asthma (Fig. 1) [6]. Because of this, inhaled londamocitinib is expected to impact on inflammatory mechanisms that are both responsive and resistant to corticosteroids, whilst minimising the potential systemic adverse effects associated with non-selective, oral JAK inhibitors [7].

A phase 2a clinical trial (AJAX, NCT06020014 [ClinicalTrials.gov], registration date 25th August 2023) is currently in progress assessing the clinical efficacy of londamocitinib in a broad population of patients with moderate-to-severe asthma. In parallel with this study, the ARTEMISIA (**A**i**R**way **T**arget **E**ngagement and **M**echanistic **I**nsight**S** of Janus kinase **I**nhibition in **A**sthma) study (NCT06435273) has been designed to obtain a deeper understanding of the mechanism of action of londamocitinib, evaluate the impact upon asthma-related pathways it may inhibit, and holds the potential to identify putative precision medicine biomarkers to guide asthma therapy [8]. In addition to sampling the airway directly, the ARTEMISIA study includes a parallel nasal and peripheral bio-sampling strategy that is shared with the AJAX study, providing a unique link between bronchial assessed mechanisms and clinical outcomes.

## Methods/design

### Study design

**Fig. 2:**
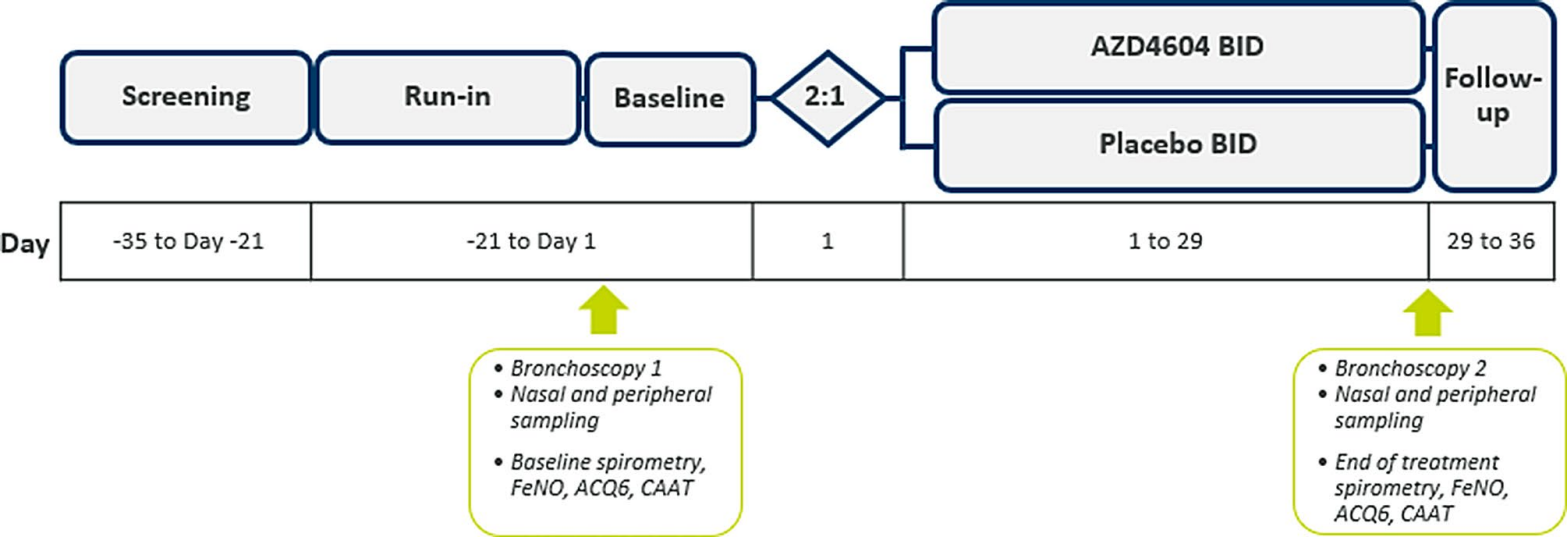
ARTEMISIA study design. Figure to illustrate the design of the ARTEMISIA study. Bronchoscopies will be undertaken to take endobronchial brushings and biopsies. Nasal sampling will consist of nasal lining fluid (nasosorption) and nasal cell (curettage) sampling. Peripheral sampling will consist of venous blood and urine. ACQ: Asthma Control Questionnaire; BID: Twice daily; CAAT: Chronic Airways Assessment Test; FeNO: Fractional exhaled nitric oxide

ARTEMISIA is a randomised, double-blind, placebo-controlled, parallel-group, exploratory, phase 2 study (NCT06435273). It aims to assess the effect of londamocitinib, administered twice daily via dry-powder inhalation for four weeks, on the wide spectrum of inflammatory signalling pathways that drive pathology in adults with moderate-to-severe asthma. The ARTEMISIA study is currently ongoing across 5 countries (Canada, Denmark, Germany, Spain, and the United Kingdom [UK]) and aims to enrol 48 participants, randomised 2:1 to londamocitinib or placebo respectively (see Fig. 2). Participants will be stratified based on fractional exhaled nitric oxide (FeNO) (≥ or < 25 parts per billion [ppb]); with at least 40% of randomised participants with FeNO ≥ 25 ppb and at least 40% with FeNO < 25 ppb at baseline.

### Inclusion criteria and recruitment

Participants eligible for the study will be aged 18–75 years and be receiving medium- or high-dose ICS and LABA with or without additional controller medication (e.g. leukotriene receptor antagonist or long-acting muscarinic antagonist) for at least 2 months prior to Visit 1 (screening) and have a history of either at least one severe asthma exacerbation within the year prior to Visit 1 or an asthma control questionnaire 6 (ACQ-6) score ≥ 1.5 during screening and prior to randomisation. Asthma patients using maintenance oral corticosteroids, systemic biologics, or ICS + fast-acting β2 agonist as a rescue medication (maintenance and reliever therapy or anti-inflammatory reliever therapy) are not eligible for the study. Key inclusion and exclusion criteria are summarised in Table 1. For the duration of the study, participants will continue to use their asthma maintenance therapy as prescribed, without change. Participants are also allowed to use short-acting β_2_ agonists as rescue medication.

**Table 1:** Key inclusion and exclusion criteria for ARTEMISIA

**Key inclusion criteria**
• Male or female, aged 18–75 years
• Body weight ≥ 40 kg and body mass index < 35 kg/m^2^
• Documented physician-diagnosed asthma for ≥ 12 months prior to visit 1
• Physician-prescribed asthma controller medication with medium- or high-dose ICS for ≥ 2 months before visit 1 (as per GINA 2023 guidelines) [9]
• Predicted normal morning pre-bronchodilator FEV_1_ ≥ 60% at visit 1 and visit 3a
• Documented evidence of asthma in previous 5 years prior to visit 1 or during screening.
• Either a history of a severe asthma exacerbation within the year prior to visit 1 or an ACQ-6 score ≥ 1.5 at Visit 1 and Visit 3a
**Key exclusion criteria**
• Clinically important pulmonary disease other than asthma
• A severe asthma exacerbation within 8 weeks prior to Visit 1
• Any disorder or major physical impairment that is not stable in the opinion of the investigator and could affect the safety of the participant throughout the study
• Any clinically significant cardiac or cerebrovascular disease
• History of venous thromboembolism, malignancy, or HIV infection
• Evidence of infective hepatitis
• Current smokers or patients with a smoking history of ≥ 10 pack-years
• Treatment with marketed biologics within 6 months or 5 half-lives of Visit 1
• Treatment with ICS plus fast-acting β2 agonist as a rescue medication (e.g., Maintenance and Reliever Treatment) is not allowed 30 days prior to Visit 1.
• Treatment with systemic corticosteroid (short-term or maintenance) use within 8 weeks (oral) or 12 weeks (intramuscular) before Visit 1.

Table detailing the key inclusion and exclusion criteria for the ARTEMISIA study. ACQ: Asthma Control Questionnaire; FEV_1_: GINA: Global Initiative for Asthma; HIV: Human immunodeficiency virus; ICS: Inhaled corticosteroids

The study consists of a screening period, 3-week run-in and baseline period, 4-week treatment period, and 1-week follow-up period (see Fig. 2).

### Objectives and outcome measures

**Table 2:** Objectives and endpoints for ARTEMISIA

Objective	Endpoint(s)
**Primary objective**	
Evaluate the effect of londamocitinib compared to placebo on gene expression in airway epithelial cells	Change from baseline to end of treatment in expression of T2, non-T2, and JAK1-related genes and gene signatures in bronchial brushings
**Secondary objectives**	
Evaluate the effect of londamocitinib compared to placebo on STAT phosphorylation in the airways	Change from baseline to end of study drug administration in STAT phosphorylation in bronchial biopsies
Explore the effect of londamocitinib on cellular pathology in the airways compared to placebo	Change in number of airway cells, including but not limited to inflammatory cells, airway smooth muscle cells, and goblet cells from baseline to end of treatment in bronchial biopsies (cells per mm² determined by microscopic evaluation)
**Key Exploratory Objectives**	
Explore if the specific inflammatory phenotypes (e.g., T2 and non-T2) in the lung are reflected in the nose and blood	Correlation of inflammatory phenotype-specific bronchial epithelial gene signatures and biomarkers with signatures and biomarkers from peripheral samples (including but not limited to blood and nasal samples)
Explore the effect of londamocitinib on biomarkers of inflammation and asthma, investigate biomarkers for predicting response to londamocitinib, evaluate exploratory biomarkers in asthma, and evaluate systemic impact on peripheral samples	The change from baseline to end of treatment in exploratory biomarkers

Table detailing the primary, secondary and key exploratory endpoints for the ARTEMISIA study. JAK: Janus kinase; STAT: signal transducer and activator of transcription; T2: type-2

The primary, secondary and key exploratory objectives and endpoints for this study are listed in Table 2. The primary objective of the ARTEMISIA study is to explore the effect of londamocitinib as compared to placebo on gene expression in airway epithelial cells. This will be assessed as a change from baseline to 4-weeks of treatment in expression of T2, non-T2, and JAK1-related genes and gene signatures in bronchial brushings. A secondary objective is to evaluate the effect of londamocitinib as compared to placebo on STAT phosphorylation in the airways. This will be assessed as change from baseline to 4-weeks of treatment in STAT phosphorylation in endobronchial biopsies. An additional secondary objective is to explore the effect of londamocitinib on cellular pathology in the airways as compared to placebo, assessed as change in number of airway cells, including but not limited to inflammatory cells, airway smooth muscle cells, and goblet cells.

**Fig. 3:**
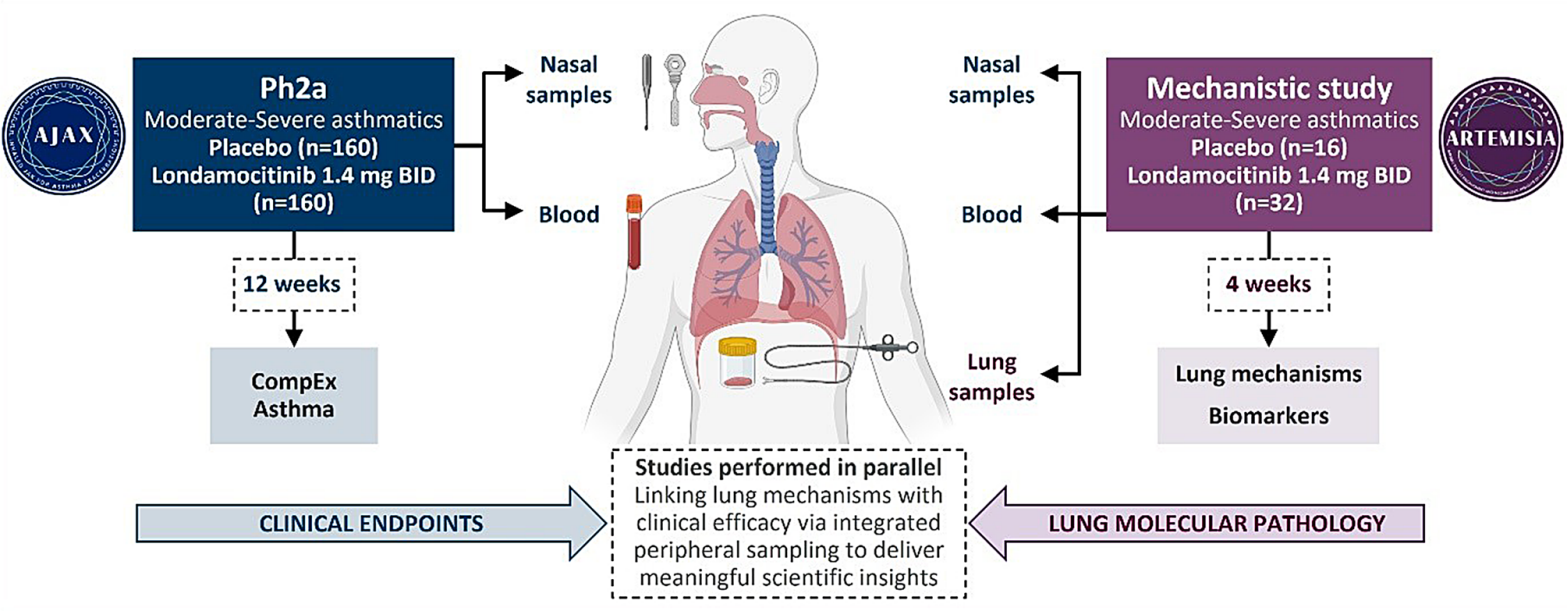
ARTEMISIA and AJAX data will be combined to relate lung sample findings with clinical outcomes. The AJAX and ARTEMISIA studies are being performed in parallel to facilitate the development of londamocitinib for moderate-severe asthma. BID: Twice daily; CompEx Asthma - Composite endpoint for asthma exacerbations [10]. Created with BioRender.com

One of our key exploratory objectives is to examine the effect of londamocitinib on biomarkers of inflammation and asthma, in order to identify biomarkers associated with a response to londamocitinib, evaluate exploratory biomarkers in asthma, and evaluate systemic impact on peripheral samples. This work will enable us to identify patterns linking changes in the central airways to tractable molecular biomarkers in peripheral samples, and subsequently combine this data with that obtained from the AJAX study to explore how these potential biomarkers relate to clinical outcomes (see Fig. 3).

### Biospecimen collection and analysis plan

**Fig. 4:**
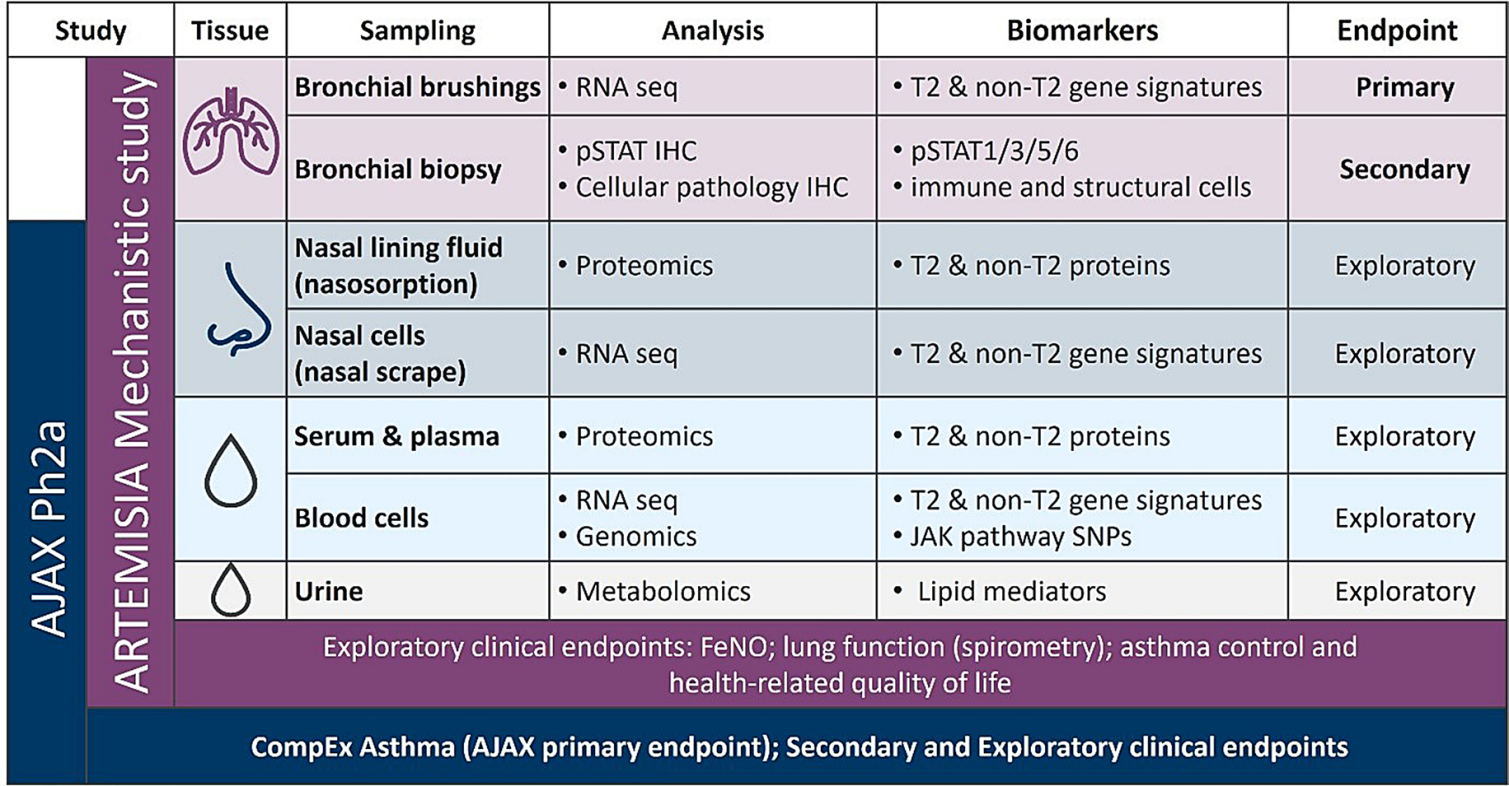
Sampling and biomarker strategy for both the ARTEMISIA and AJAX studies. Molecular biomarkers in more easily accessible matrices, such as nasal samples, blood, and urine will be correlated to T2 and non-T2 bronchial epithelial gene signatures amenable to londamocitinib modulation. Complementary precision sampling in AJAX and ARTEMISIA will enable the exploration of a link between lung airway mechanisms and clinical efficacy to identify biomarkers predictive of a response to londamocitinib and inform patient subgroups in asthma. CompEx Asthma - Composite endpoint for asthma exacerbations [10]; FeNO: Fractional exhaled nitric oxide; IHC – immunohistochemistry; pSTAT– phosphorylated signal transducer and activator of transcription; RNA: Ribonucleic acid; Seq: Sequencing; SNP: Single nucleotide polymorphism; T2: Type-2

Paired lung, nasal, and peripheral samples will be collected prior to the first dose at Visit 3a and again at the end of the treatment period at Visit 6a (Figs. 2 and 4). Whole blood, serum, and plasma will be collected, processed per standard protocols, and stored for exploratory transcriptomics and proteomics analysis. Urine will be collected and immediately frozen without additives for exploratory metabolomics analysis. Nasal mucosal lining fluid will be collected via nasosorption from each nostril and stored for exploratory proteomics analysis. Nasal curettage will then be performed for each nostril and cellular material combined into a single tube containing RNAlater solution and cryopreserved for bulk ribonucleic acid (RNA) sequencing at the end of the study.

**Fig. 5:**
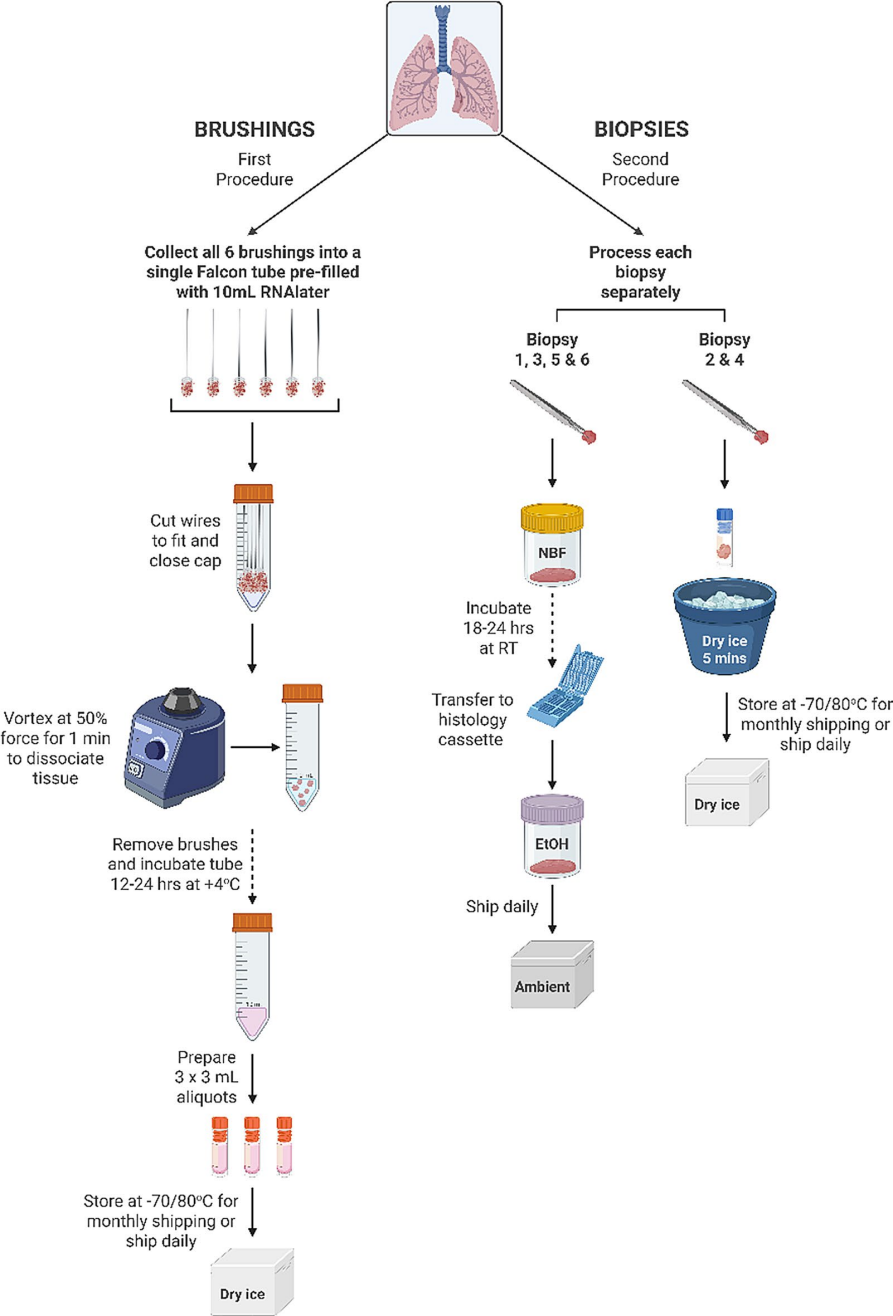
Processing of bronchoscopic samples. Highly prescriptive sample acquisition and handling protocols were provided to ensure sample handling was consistent across the sites. Created with BioRender.com

Endobronchial brushings and biopsies will be collected via bronchoscopy following local guidelines. The aim will be to collect a total of 6 endobronchial brushings followed by 6 endobronchial biopsies from the 2nd to 5th order carinae of the right lung. All six brushing samples will be collected in a single tube containing RNAlater solution and cryopreserved for bulk RNA sequencing at the end of the study. Four of the biopsies will be fixed in neutral buffered formalin and embedded in paraffin for analysis of cellular pathology and STAT phosphorylation by immunohistochemistry. Two of the biopsies will be immediately snap frozen and stored for proteomics analysis (Fig. 5).

To ensure uniformity of sample acquisition and handling processes, detailed protocols describing these procedures were provided to sites, alongside comprehensive training sessions.

### Biomarker inclusion for primary endpoint

The primary endpoint of ARTEMISIA is designed to assess the proposed broad anti-inflammatory effects of londamocitinib on Th1, Th2, Th17, and IL-6 trans signalling pathways. As such, we have pre-defined genes and gene sets reporting on the different inflammatory pathways, which will be interrogated in the transcriptomic data sets generated from bronchial brushings pre- and post-treatment with londamocitinib or placebo. These genes and gene sets were selected based on integrating in-house data obtained through the *in vitro* treatment of human bronchial epithelial cell lines with relevant cytokine stimuli plus or minus londamocitinib along with published data derived from lung samples from patients with asthma [11–14]. Our analysis plan for the primary endpoint will follow a similar approach used in our previous study, in which the IL-6 trans signalling signature was derived and used to interrogate expression of this pathway in lung brushings from patients with asthma [15].

In brief, our strategy for analysing individual genes will be to consider a 20%-fold change in either direction from baseline to post-treatment to be the minimally interesting effect, depending on what is expected for the individual gene. The average fold change will then be compared between the londamocitinib and placebo arms. For gene signatures, gene set variation analysis (GSVA) and single-sample scoring (singscore) will be used to compare changes between the two treatment arms.

### Statistical considerations

All statistical analyses of primary, secondary and exploratory outcomes will be performed after database lock, which will occur after the last subject last visit study milestone.

Data on patient demographics and disease characteristics, as well as safety findings, will be reported descriptively for the full analysis set (all randomised participants who receive at least one dose of study drug irrespective of protocol adherence and continued participation in the study).

Due to the exploratory nature of the study, no formal power calculation was performed. The sample size of 48 participants was estimated based on a large sample z-statistic around the difference in change from baseline values between londamocitinib and placebo on the logarithmic scale. A pooled standard deviation for change from baseline across T2 genes was derived from a historical bronchoscopy study [13]. Assuming comparable variability, the estimated sample size will ensure that the two-sided 95% confidence intervals for the biomarkers of interest will not be wider than 0.72 on the log-change from baseline scale, which is considered sufficiently narrow for the purposes of this study.

The bulk RNA-sequencing data collected for the primary endpoint will be analysed using negative-binomial regression models designed for count-based gene expression data [16, 17]. Variations in sequencing depth between samples will be modelled, along with adjustment for study drug as a covariate. A natural-logarithm link function will be used for the generalised linear model, but results will be presented in terms of a log-2-fold change. The treatment effect on gene-expression change from baseline will be analysed in a paired fashion.

The secondary endpoints aim to evaluate the effect of londamocitinib compared to placebo on various bronchial biopsy measures throughout the study and will be analysed using analysis of covariance (ANCOVA) models. The within-participant change from baseline to end of treatment measures of STAT phosphorylation and airway inflammatory cells from the bronchial biopsies will be modelled individually on the log-scale and will include baseline value and study drug as covariates.

The exploratory endpoints will further describe biomarker effects and summarise the effect of londamocitinib on lung function and asthma characteristics throughout the study compared to placebo. The analysis of additional biomarker expression values will follow similar methods as in the primary endpoint analysis. Change from baseline spirometry values will be analysed using Mixed Model for Repeated Measures (MMRM) methods. Additional asthma disease characteristics and pharmacokinetic measures will be summarised descriptively and with graphical displays.

## Discussion

In the Phase 1 study (NCT04769869), inhaled londamocitinib was shown to have acceptable safety and tolerability, whilst also demonstrating the ability to significantly reduce FeNO in those with mild asthma [7]. This study provided evidence of inhibition of T2 inflammatory pathways in the absence of systemic target engagement by londamocitinib. The phase 2 ARTEMISIA study is designed to provide evidence of JAK1 target engagement directly in the lung, assess impact of londamocitnib on non-T2 inflammatory pathways, and explore additional molecular and cellular changes observed in the lung. In addition, this study will assess how those changes are reflected in peripheral samples from the nose, blood and urine.

The primary objective of ARTEMISIA is to assess the change in epithelial gene signatures of central airways following 4 weeks of exposure to londamocitinib. Many studies investigating the impact of drugs on the airways have focussed on the histopathological changes seen in the airway [18, 19]. Local effects of blocking IL-13 signalling on eosinophilic airway inflammation in moderate-severe asthmatics was investigated in the MESOS and CLAVIER studies [19, 20]. In these studies, neither tralokinumab nor lebrikizumab treatment resulted in a significant effect on change from baseline in airway submucosal eosinophils versus placebo. It should be noted however that enrolment to the CLAVIER study was stopped before inclusion of the planned number of participants. Inhibition of TSLP signalling in the CASCADE study did result in a significantly greater change from baseline to the end of treatment in airway submucosal eosinophils with tezepelumab compared to placebo (ratio of geometric least-squares means 0·15 [95% CI 0·05–0·41]; nominal *p* < 0·0010) [18]. To adequately assess these changes requires prolonged exposure to the drug and substantial cohorts, leading to large, lengthy studies which can be difficult to recruit to and lead to incomplete datasets [20]. Using gene expression as our primary endpoint allows us to evaluate inflammatory changes whilst reducing both duration of exposure to study drug and sample size for the study. This not only allows us to evaluate mechanism of action in a more efficient way, but also allows us to address the ethical imperative of reducing the number of participants involved in an invasive study, particularly at this early stage of drug development. Whilst the primary objective of the ARTEMISIA study is to assess the change in epithelial gene signatures, the study will also assess the impact of inhibiting the IL-13, IL-4, IL-6, IFN-γ and TSLP signalling pathways on cellular pathology as a secondary objective.

Performing studies with complex sampling methods at scale increases the risk of logistical challenges and variation in sample collection and quality which may lead to a lack of veracity of the data that is collected. Given the risks and complexity, to perform bronchoscopy on participants at a scale which would allow the data collected to be related directly to clinical outcomes would be resource intensive and difficult to deliver. Identifying a relationship between central and peripheral biomarkers which can then be related to clinical outcomes in a separate large-scale study offers a solution to this problem. Evaluating epithelial gene signatures has allowed us to keep the sample size small, reducing possible site-to-site sampling variability and logistical complexity. By collecting the same peripheral samples in both the ARTEMISIA and AJAX studies, associations between biomarkers in central and peripheral samples will be evaluated, and subsequently related to clinical outcomes, without needing to expose large numbers of participants to the risks associated with invasive sampling.

Randomisation in the ARTEMISIA study will be stratified based on baseline FeNO as a non-invasive approach to enrol participants across the spectrum of T2- and T17-driven airway inflammation. FeNO level has previously been reported to be positively correlated with T2 gene expression signatures and inversely correlated with T17 gene expression in airway epithelial cells [13].

Key barriers to performing studies of this nature include participant acceptability but also feasibility of delivery at the site level. To address these challenges, the study protocol was reviewed and critiqued by a group of asthma patients, as well as a group of research nurses from the UK NIHR Clinical Research Facility Network. The study was also reviewed and subsequently adopted by the NIHR translational research collaboration. By incorporating the perspective of participants and research delivery staff, the authors hope to have created a study design which achieves its objectives but remains acceptable and feasible to deliver from the outset.

There are limitations to our study design. Whilst the sample size has intentionally been kept to a minimum for the reasons set out above, this may limit data interpretation, particularly when attempting to assess the effect of londamocitinib in T2 and non-T2 predominant asthma. ARTEMISIA is a hypothesis-generating study designed to provide insights to inform the future clinical development plans for londamocitinib. Another limitation is that due to the short duration of exposure to study drug and small sample size, meaningful changes in clinical effects are not anticipated. This is mitigated for by collecting the same nasal, blood and urine samples in the much larger AJAX study which is designed to investigate clinical outcome measures and is exposing 320 participants to study drug for 12 weeks.

## Conclusion

There is a large unmet need for new treatments in asthma which target non-T2 inflammatory pathways. ARTEMISIA will evaluate the effect of londamocitinib, a novel JAK-1 inhibitor, on gene expression in the airways of a broad population of uncontrolled, moderate-to-severe asthma with varying degrees of T2 inflammation. ARTEMISIA not only aims to assess the impact of londamocitinib on T2 and non-T2 inflammatory pathways, but also delineate the relationship between the central and peripheral airway, and potentially identify novel biomarkers assessed with minimally invasive sampling methods that can be used to identify those with non-T2 inflammation. Whilst the participants taking part in ARTEMISIA all have asthma, insights gained from this study may inform future work assessing the utility of JAK1 inhibition in other respiratory diseases.

## Data Availability

No datasets were generated or analysed during the current study.

## Abbreviations

ACQ: Asthma Control Questionnaire
ANCOVA: Analysis of covariance
BID: Twice daily
BMI: Body mass index
CAAT: Chronic Airways Assessment Test
Compex Asthma: Composite endpoint for asthma exacerbations
FeNO: Fractional exhaled Nitric Oxide
FEV_1_: Forced expiratory volume in 1 second
GINA: Global Initiative for Asthma
GSVA: Gene set variation analysis
HIV: Human immunodeficiency virus
ICS: Inhaled corticosteroids
IHC: Immunohistochemistry
IFN: Interferon
IL: Interleukin
JAK: Janus kinase
LABA: Long-acting beta agonists
LAMA: Long-acting muscarinic antagonist
LTRA: Leukotriene receptor antagonist
MMRM: Mixed model for repeated measures
PPB: Parts per billion
RNA: Ribonucleic acid
Seq: Sequencing
Singscore: Single-sample scoring
SNP: Single nucleotide polymorphism
STAT: Signal transducer and activator of transcription
T2: Type 2
Th: T-helper
TSLP: Thymic stromal lymphopoietin
UK: United Kingdom
